# *MAML2* Rearrangement in Primary Pulmonary Mucoepidermoid Carcinoma and the Correlation with FLT1 Expression

**DOI:** 10.1371/journal.pone.0094399

**Published:** 2014-04-08

**Authors:** Fen Zhu, Weige Wang, Yingyong Hou, Jindong Shi, Zilong Liu, Deming He, Chunxue Bai, Shanqun Li, Liyan Jiang

**Affiliations:** 1 Department of Pulmonary Medicine, Zhongshan Hospital, Fudan University, Shanghai, China; 2 Department of Pathology, Fudan University Shanghai Cancer Center, Shanghai, China; 3 Department of Pathology, Zhongshan Hospital, Fudan University, Shanghai, China; 4 Department of Pulmonary Medicine, Shanghai Chest Hospital, Shanghai Jiaotong University, Shanghai, China; 5 Department of Oncology, Shanghai Medical College, Fudan University, Shanghai, China; 6 The Fifth People's Hospital of Shanghai, Fudan University, Shanghai, China; Texas Tech University Health Sciences Center, United States of America

## Abstract

**Introduction:**

Primary pulmonary mucoepidermoid carcinoma (PMEC) is an uncommon neoplasm with remarkable resemblance to mucoepidermoid carcinoma of the salivary glands. The latter has been shown to harbor t(11,19) resulting in *MECT1-MAML2* fusion, which may be of diagnostic and prognostic values. However, the importance of such feature in PMEC has not been well studied.

**Methods:**

We detected *MAML2* rearrangement using fluorescence *in situ* hybridization (FISH) in tissue samples from 42 cases of PMEC and 40 of adenosquamous carcinoma (ASC), and the expression of potential downstream targets of *MECT1-MAML2*, including HES1, FLT1 and NR4A2 with immunohistochemistry (IHC). The findings were then examined regarding the clinicopathological parameters and patient outcomes.

**Results:**

FISH analysis revealed *MAML2* rearrangement in 50% of the PMEC cases, and such property was prominent in considerable younger patients (33 versus 60 years; *p* = 0.001) and restricted to cases of low and intermediate grades. IHC analysis showed that FLT1 and HES1 were expressed at lower level in *MAML2* rearranged group than *MAML2* non-rearranged group (*p*<0.001 and *p* = 0.023, respectively). Survival analysis showed significant correlation between *MAML2* rearrangement and overall survival (*p* = 0.023) or disease-free survival (*p* = 0.027) as well as correlation between FLT1 and overall survival (*p* = 0.009).

**Conclusions:**

*MAML2* rearrangement appears frequent in PMEC and specific with this tumor. Both the presence of *MAML2* rearrangement and absence of FLT1 tend to confer a favorable clinical outcome. These findings suggest that molecular detection of *MAML2* rearrangement combined with FLT1 may be of important clinical value for PMEC.

## Introduction

Primary pulmonary mucoepidermoid carcinoma (PMEC), an uncommon malignancy, generally derives from minor salivary glands of tracheobronchial tree [1]. It is morphologically similar to mucoepidermoid carcinoma arising from salivary glands of the head and neck, and poses diagnostic challenge with common lung cancers, specifically, adenosquamous carcinoma (ASC) due to their morphologic mimics. Clinicopathological parameters, such as age, stage and grade are the most significant prognostic factors of PMEC [2]–[4]. Low-grade PMECs usually impart an indolent clinical course, whereas high-grade tumors result in poor prognosis. However, current grading systems seem in deficiency on exclusive basis to subjectively assess diverse histological parameters for determining either low-, intermediate- or high-grade tumors. In addition, grading systems used have been hindered by poor reproducibility and variability between different systems particularly with respect to intermediate grade [5]–[6]. Therefore, these limitations can be compromised by introducing molecular markers that shall be more objective and are desirable in stratifying patients into appropriate treatment groups.

Earlier studies indicated that the t(11;19)(q21;p13) resulting in gene fusion of mucoepidermoid carcinoma translocated 1–mammalian mastermind like 2 (*MECT1-MAML2*) is the primary chromosomal abnormality observed in mucoepidermoid carcinoma of the head and neck [7]–[10]. *MECT1-MAML2* fusion consists of CREB-binding domain of *MECT1* fused to the transactivation domain of the Notch co-activator *MAML2* [8], [9], and may facilitate to activate both Notch signaling target genes and cAMP/CREB target genes, inducing independent cell proliferation and differentiation function [8], [9], [11], [12]. The incidence of *MECT1-MAML2* fusion varies somewhat in mucoepidermoid carcinomas. Nevertheless, it is generally accepted that 38–81% tumors manifest this fusion [13]. This fusion is observed to confer a favorable prognosis and also thought to be fairly specific for mucoepidermoid carcinoma of the salivary glands [7], [10], . Despite the morphological similarity of PMEC to its salivary glands counterpart, it remains unknown the precise frequency of *MAML2* gene rearrangement and its clinicopathological implications in PMEC.

In an effort to estimate the prognostic value of *MAML2* rearrangement in refining clinicopathological prognostic factors in PMEC and determine its potentiality in discriminating PMEC from morphologic mimics, we detected the prevalence of the rearrangement nature of *MAML2* by using fluorescence *in situ* hybridization (FISH) in tissue samples obtained from 42 cases of PMEC and 40 of ASC. To examine the molecular consequences of such feature, we also detected the expression of potential downstream targets of the *MECT1-MAML2* fusion, including Notch target (HES1) and cAMP/CREB targets (FLT1 and NR4A2).

## Materials and Methods

### Cases and tissue samples

The study was conducted under the approval by the Institutional Review Board. Ethical approval was granted by the Clinical Research Ethics Committee of Zhongshan Hospital, Fudan University (Shanghai, China). And signed informed consent was obtained from all included patients for the acquisition and use of tissue samples and anonymized clinical data. Tissue samples of formalin-fixed, paraffin-embedded (FFPE) blocks in 42 cases with primary PMEC were available from Zhongshan Hospital and Shanghai Chest Hospital between 2004 and 2011. Another 40 specimens of primary ASC cases were obtained from Zhongshan Hospital during 2007 and 2011. Hematoxylin and eosin (H&E)-stained slides in all cases were reviewed independently by two experienced pathologists (WW and YH). And any disagreement was submitted to other pathologists to achieve a consensus. Diagnosis of PMEC and ASC was carefully made according to the World Health Organization (WHO) classification of thoracic tumors [1]. PMECs were graded in line with the algorithm proposed by Auclair et al [16]. Briefly, grading was based on a points system: intracystic component >20%, 2 points; neural invasion, 2 points; necrosis, 3 points; four or more mitoses per 10 high-power fields, 3 points; anaplasia, 4 points; A total score between 0 and 4 defines a low-grade tumor, a score of 5 to 6 applies to an intermediate-grade tumor, and a score of 7 or more indicates a high-grade tumor. For the diagnosis of ACS in our series, all the following parameters should be met: 1) the squamous cell carcinoma components showing unequivocal keratin or intra-cellular bridges; 2) adenocarcinoma components positive for TTF-1 and/or PE 10 and/or Napsin-A stainings; and 3) components of both squamous cell carcinoma and adenocarcinoma with each comprising at least 10% of the tumor. Additionally, in order to make the classification of high-grade PMEC and ASC clear, a more rigid definition of high-grade PMEC was added in this study. Specially, high-grade PMEC included should meet the following criteria: 1) located centrally or endobronchial and exophytic growth pattern; 2) no keratinisation pearls; and 3) negative for TTF-1 and/or PE 10 and/or Napsin-A stainings. Demographic and clinical parameters were noted including age, sex, tumor size, nodal status, intrathoracic invasion, treatment, recurrence, metastasis and outcome. Staging was performed in compliance with the tumor-node-metastasis (TNM) staging system of American Joint Committee on Cancer (7th edition). Follow-ups were obtained via either medical records or telephone interview. FFPE blocks, which were taken from archival cases of primary PMEC and primary lung ASC, were sectioned at a thickness of 4-μm.

### Fluorescence *in situ* hybridization (FISH) analysis

FFPE tissue sections of PMECs and ASCs were tested by FISH to detect the rearrangement of *MAML2* gene at 11q21 locus. FISH was performed using *MAML2* Dual Color Break Apart Probe (ZytoVision, Bremerhaven, Germany) according to the protocol recommended by the manufacturer of FISH-Tissue Implementation Kit (ZytoVision, Bremerhaven, Germany). Briefly, the sections were deparaffinized in xylene, dehydrated with ethanol, pretreated in pretreatment buffer for 15 minutes, and then incubated with pepsin solution for 10 minutes. After denaturation at 75°C for 10 minutes, the sections were incubated with probe for 24 hours at 37°C in hybridization oven (Dako, Glostrup, Denmark). Post-hybridization washes were carried out twice for 5 minutes at 37°C. The sections were air-dried, protected from light and counterstained with DAPI/Antifade-Solution.

A signal pattern consisting of 1 green/orange fusion signal indicated a normal 11q21 locus, one separate green and one separate orange signal indicated translocation of the 11q21 locus (split signal). Primary salivary mucoepidermoid carcinoma cases known to harbor t(11;19)(q21;p13) were taken as the positive control. For the negative control and development of cut-off value of *MAML2* rearrangement, 10 cases of normal parotid tissue and 10 cases of normal bronchus were investigated, respectively. In accordance with the controls and the algorithm described previously [17], 10 split signals per 100 nuclei counted was considered as a positive result of *MAML2* rearrangement in current study.

### Immunohistochemistry (IHC) study

The expression of several potential downstream targets of the *MECT1-MAML2* fusion, including the Notch target (HES1) and the cAMP/CREB targets (FLT1 and NR4A2) were analyzed using IHC on 4 μm-FFPE sections of PMEC with two-step method (EnVision Detection Systems, Dako, Glostrup, Denmark) according to the manufacturer's protocol. Sections were sequentially deparaffinized in xylene three times for 10 minutes each round, dehydrated with 100%, 90% and 70% ethanol, heated in an autoclave with pH = 8.0 EDTA buffer at 120°C for 5 min for antigen retrieval, and incubated overnight with primary antibodies for anti-HES1, anti-FLT1 or anti-NR4A2 (Epitomics, California, USA), which were respectively diluted 1∶200, 1∶300 or 1∶150, in a moist chamber at 4°C. Then, the sections were rinsed with PBS and incubated again in secondary antibody for 30 minutes at 37°C. The antigen-antibody complex were visualized with 3,3′-diaminobenzidine solution.

H-score [18] was used for semiquantitative analysis of immunoreactivity of HES1, FLT1 and NR4A2. The score was obtained as the formula: 3× percentage of strongly staining +2× percentage of moderately staining +1× percentage of weakly staining, giving a range of 0 to 300. Positive immunoreactivity was defined as H-score >0. Score was independently obtained by two of the authors (WW and DH).

### Statistical analyses

Categorical variables were presented as counts and percentages, and comparisons were conducted using Fisher's exact test. Continuous variables were presented as median and range, and comparisons were performed using Mann-Whitney test or Kruskal-Wallis test. Spearman correlation and linear regression was used for H-score. Survival was calculated using the Kaplan–Meier method, and the survival curves were compared by the log-rank test. Cox's proportional hazards regression model was used for multivariate survival analysis. Two-tailed *P* value<0.05 was regarded as statistically significant. All analyses were performed using GraphPad Prism version 5.0 (GraphPad Software, San Diego California, USA), and multivariate survival analysis was performed using IBM SPSS Statistics 20.0 (IBM Corporation, New York, NY, USA).

## Results

### Clinical and pathological findings in PMEC cases

Of the 42 PMEC cases, 25 were males and 17 females. The median age was 53.5 years (range 14–76 years). Twenty-six cases were disease of stage I, 10 stage II, 5 stage III and 1 stage IV, respectively. Histopathological examination of PMEC showed that 23 cases presented with low-grade tumors, 10 were intermediate-grade and 9 high-grade. Apart from one perioperative death, the remaining patients were followed up postoperatively. The median follow-up duration was 57 months (range 2–117 months), during which the tumor recurrence/metastasis was identified in 9 patients. Six tumor-related deaths occurred.

### FISH findings

**Figure 1:**
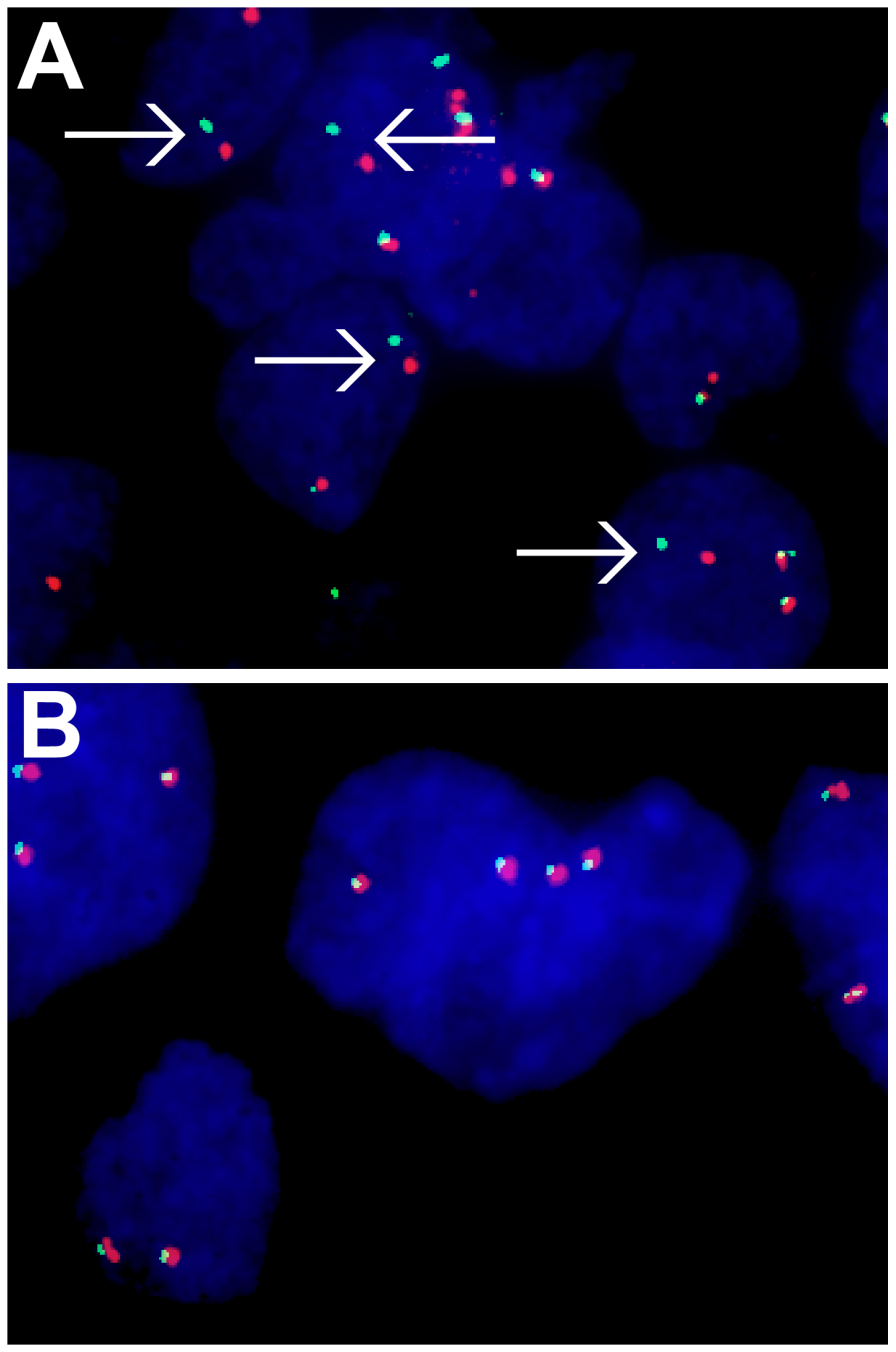
FISH analysis for *MAML2* gene. Arrows indicate spit signals showing the rearrangement of *MAML2*. *A*, Case of pulmonary mucoepidermoid carcinoma. *B*, Case of adenosquamous carcinoma.

FISH evaluation was successfully performed in all cases. Twenty-one of 42 (50%) PMEC tumors showed positive *MAML2* rearrangement (Figure 1A). Histological distribution in the 21 PMEC cases with *MAML2* rearrangement was associated with grade of low (15/23) intermediate (6/10) and high (0/9) compared to those without rearranged *MAML2* by grade of low (8/23), intermediate (4/10) and high (9/9). Comparison of the scales among the three groups showed that disease with *MAML2* rearrangement was significantly rest on a lower pathological grade (*p* = 0.002). The total 40 ASC tumors were negative for *MAML2* rearrangement (Figure 1B).

### Immunohistochemical findings

**Figure 2:**
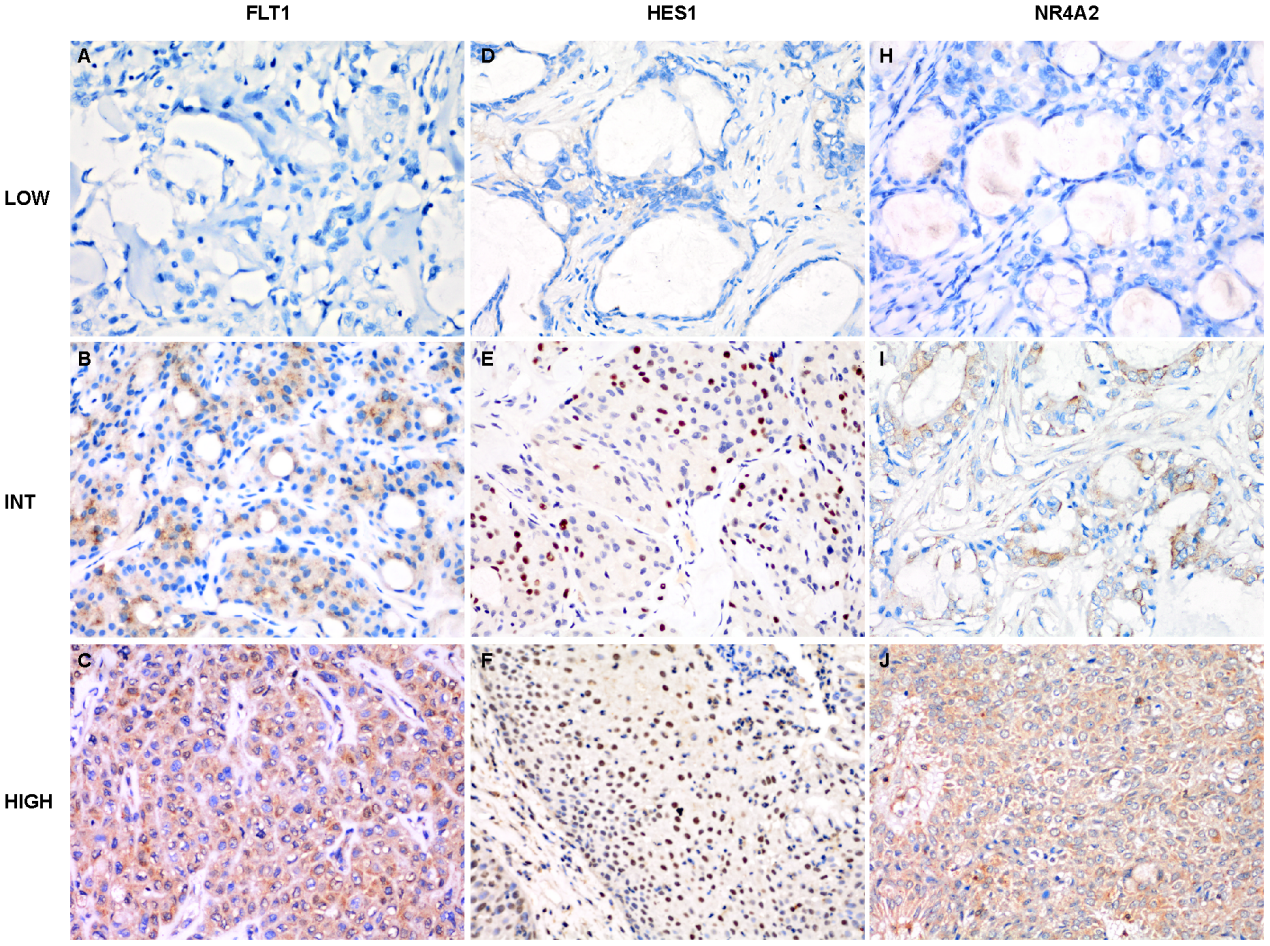
Immunostaining for FLT1, HES1 and NR4A2 in pulmonary mucoepidermoid carcinomas (400×). *A–C*, Immnostaining for FLT1 in low (*A*), intermediate (*B*) and high (*C*) grades of tumors. *D–F*, Immnostaining for HES1 in low (*D*), intermediate (*E*) and high (*F*) grade tumors. *G–I*, Immnostaining for NR4A2 in low (*G*), intermediate (*H*) and high (*I*) grade tumors.

**Figure 3:**
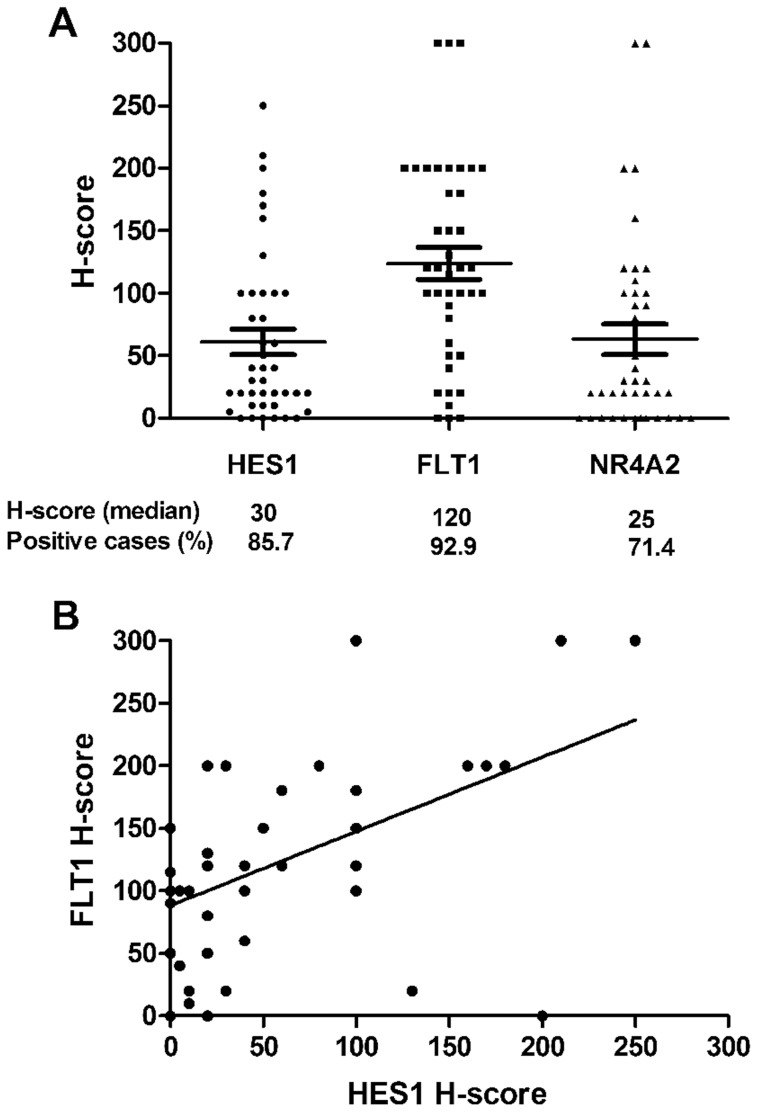
Scattered plots showing H-score of HES1, FLT1 and NR4A2 in pulmonary mucoepidermoid carcinomas. *A*, Variation of the H-score for HES1, FLT1 and NR4A2; The median H-score for each target and the percentage of positive cases are also shown. *B*, Correlation between the H-score for HES1 and FLT1. A significant correlation (r = 0.463; *p* = 0.003) was detected.

The expression of HES1, FLT1 and NR4A2 was examined by immunohistochemical staining, and positive immunoreactivity was detected in the majority of PMEC cases besides variation in the percentage of positive cells as well as intensity existing among low-, intermediate- and high-grade tumors (Figure 2A–J). Kruskal-Wallis test revealed that H-score for HES1 and FLT1 were significantly associated with higher pathological grade (*p* = 0.014 and *p* = 0.009, respectively). The number of positive cases and the H-score value (median and range) in PMEC tumors were as follows: HES1, 36 (85.7%) and 30 [0–250]; FLT1, 39 (92.9%) and 120 [0–300]; and NR4A2, 30 (71.4%) and 25 [0–300] (Figure 3A). There was a positive correlation between the immunoreactivity for HES1 and FLT1 (r = 0.463; *p* = 0.003; Figure 3B). No significant correlation was observed between the immunoreactivity for FLT1 and NR4A2, as well as for HES1 and NR4A2.

### Correlation between *MAML2* rearrangement and immunoreactivity for HES1, FLT1 and NR4A2

**Figure 4:**
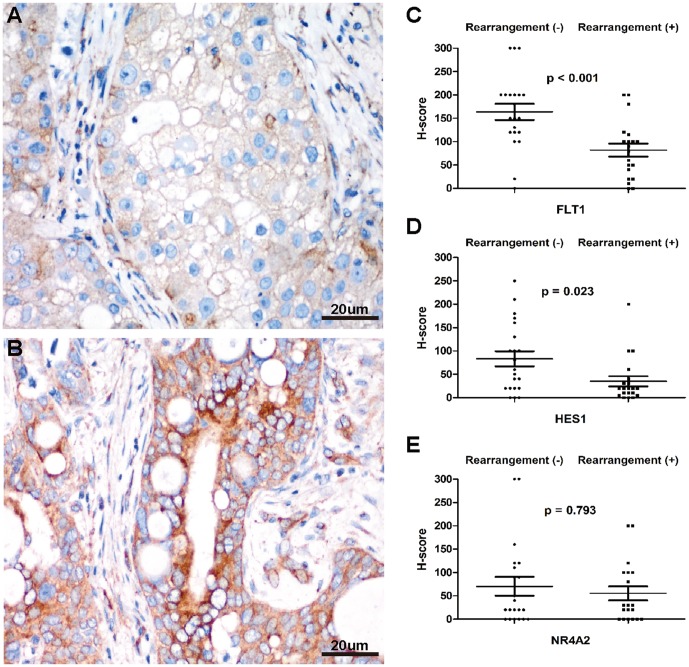
Correlation between *MAML2* rearrangement and expression of FLT1, HES1 and NR4A2 in pulmonary mucoepidermoid carcinomas. *A–B*, Immunostaining for FLT1 in *MAML2* rearranged tumor (*A*) and *MAML2* non-rearranged tumor (*B*) (400×). *C–E*, Scattered plots showing correlation between *MAML2* rearrangement and variation of the H-score for FLT1(*C*), HES1 (*D*) and NR4A2 (*E*).

Comparisons between *MAML2* rearranged PMECs and *MAML2* non-rearranged PMECs revealed that the former had significantly down-regulated immunoreactivity for FLT1 (*p*<0.001; Figure 4A–C). There was also a reduction in HES1 immunoreactivity in *MAML2* rearranged tumors (*p* = 0.023; Figure 4D). No obvious difference on NR4A2 immunoreactivity was found between the two groups (Figure 4E). Percentage of positive cases for HES1, FLT1 and NR4A2 were respectively 85.7%, 95.2% and 71.4% in group negative for *MAML2* rearrangement, and 85.7%, 90.5% and 71.4% positive for *MAML2* rearrangement.

### Correlation between *MAML2* rearrangement and clinicopathological parameters in PMEC patients

Table 1 shows the correlation between the clinicopathological parameters and *MAML2* rearrangement in PMEC patients. Compared with cases without *MAML2* rearrangement, *MAML2* rearranged cases were seen in considerable younger patients (median, 33 versus 60 years; *p* = 0.001). Diseases with *MAML2* rearrangement were within low and intermediate grades. None of *MAML2* rearranged cases was detected in high-grade tumors. There was a significantly higher frequency of *MAML2* rearrangement in low/intermediate grades than that in high grade (*p* = 0.001). No significant difference was found between *MAML2* rearranged cases and *MAML2* non-rearranged cases regarding the gender, tumor size, TNM stage, nodal status or invasion of intrathoracic structures including main bronchus, pleura, chest wall, diagram, phrenic nerve and pulmonary vessels.

**Table 1:** Clinicopathological Correlation of *MAML2* Rearrangement in PMECs.

	*MAML2* rearrangement		
Variables	Positive (n = 21)	Negative (n = 21)	*P*
Median age [range]	33 [14–73]	60 [27–76]	**0.001**
Gender (M/F)	13/8	12/9	1.000
Tumor size [range], cm	3.0 [0.5–6.5]	3.0 [0.5–10.0]	0.693
TNM stage			
I∼IIA	19 (90.5)	17 (81.0)	0.663
IIB∼IV	2 (9.5)	4 (19.0)	
LN involvement			
Yes	2 (9.5)	5 (23.8)	0.410
No	19 (90.5)	16 (76.2)	
Intrathoracic invasion			
Yes	4 (19.0)	6 (28.6)	0.719
No	17 (81.0)	15 (71.4)	
Pathological grade			
Low/Intermediate	21 (100.0)	12 (57.1)	**0.001**
High	0 (0.0)	9 (42.9)	
Recurrence/metastasis			
Yes	2 (9.5)	7 (33.3)	0.130
No	19 (90.5)	14 (66.7)	

Abbreviations: MAML2, mastermind-like gene 2; PMECs, pulmonary mucoepidermoid carcinomas; TNM, tumor-nodal-metastasis; LN, lymph node.

### *MAML2* rearrangement and FLT1 immunoreactivity correlate with prognosis in PMEC patients

**Figure 5:**
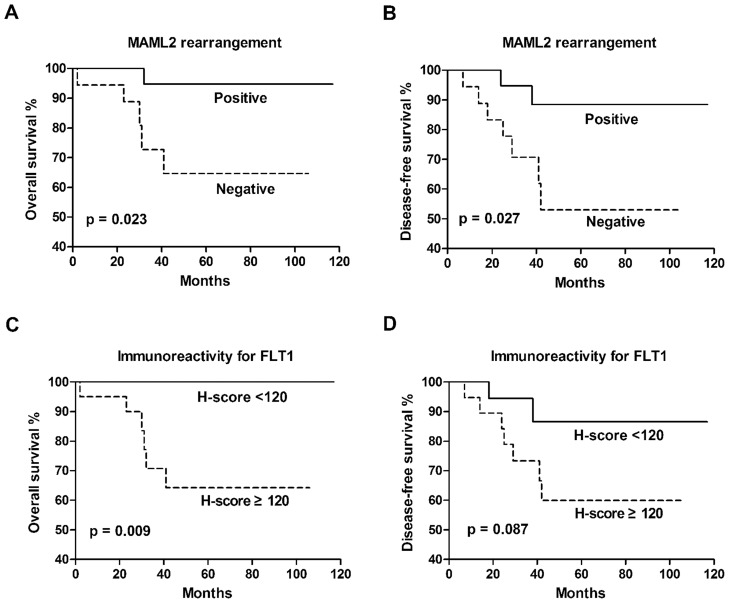
Kaplan-Meier curves for overall survival (OS) and disease-free survival (DFS) in patients with pulmonary mucoepidermoid carcinoma. *A–B*, OS (*A*) and DFS (*B*) for the *MAML2* rearrangement. *C–D*, OS (*C*) and DFS (*D*) for the immunoreactivity of FLT1.

The group of patients positive for the *MAML2* rearrangement showed significantly better overall survival (OS) (*p* = 0.023; Figure 5A) and disease-free survival (DFS) (*p* = 0.027; Figure 5B) than the negative group. OS and DFS at 5-year were 94.7% and 88.4% for the positive group compared to 64.6% and 53.0% for the negative group. Recurrence/metastasis occurred in 2 patients from the positive group and 7 of the negative group. Death was one for the positive group and 5 for the negative group.

As we chose median H-score as the cut-off value of immunoreactivity in survival analysis, the threshold H-score for FLT1, HES1 and NR4A2 was respectively set at 120, 30 and 25 in current study. We found that FLT1 immunoreactivity was in significant correlation with OS (*p* = 0.009; Figure 5C), but did not significantly correlate with DFS (*p* = 0.087; Figure 5D) in PMEC cases. No significant correlation was seen between immunoreactivity for HES1 or NR4A2 and OS or DFS.

Clinicopathological parameters including age, TNM stage, lymph node involvement and pathological grade were also found to be related with OS (*p* = 0.001, 0.03, 0.03, 0.011) and DFS (*p* = 0.01, 0.04, 0.039, 0.01). Although intrathoracic invasion was associated with OS (*p* = 0.03),yet did not have significant effect on DFS. Multivariate survival analysis of all parameters suggested that positive *MAML2* rearrangement was an independent protective factor of OS (*p* = 0.042, HR: 0.068, 95%CI: 0.008–0.614), and high pathological grade was an independent risk factor of OS (*p* = 0.037, HR: 11.706, 95%CI: 1.825–79.203) as well as DFS (*p* = 0.031, HR: 11.687, 95%CI: 2.289–59.659) in patients with PMEC (Table 2).

**Table 2:** Prognostic Factors for Survival in Multivariate Analysis.

	Overall Survival			Disease-free Survival		
Variables	*P*	HR	95%CI	*P*	HR	95%CI
Age	0.176	—	—	0.064	—	—
TNM stage	0.461	—	—	0.493	—	—
I∼IIA						
IIB∼IV						
Intrathoracic invasion	0.188	—	—	0.750	—	—
Yes						
No						
LN involvement	0.583	—	—	0.132	—	—
Yes						
No						
Pathological grade	**0.037**	11.706	1.825–79.203	**0.031**	11.687	2.298–59.659
Low/Intermediate						
High						
*MAML2* rearrangement	**0.042**	0.068	0.008–0.614	0.110	—	—
Positive						
Negative						
FLT1 expression	0.098	—	—	0.502	—	—
H-score≥120						
H-score<120						

Abbreviations: TNM, tumor-nodal-metastasis; LN, lymph node.

## Discussion

The present study explored the significance of *MAML2* rearrangement detected with FISH by using FFPE tissue sections of primary pulmonary mucoepidermoid carcinoma in a large series. And we deployed a more narrow definition of PMEC to exclude the potential ASC from high-grade PMEC. We found that *MAML2* rearrangement was presented in 50% of PMEC tumors. This prevalence is lower than the 77% described previously in a smaller series study by Achcar Rde O et al [19]. Such discrepancy may be attributed partly to the different sample size on one hand, and on the other, it may have been caused by the case inclusion, since PMEC is characterized by wide variation in histology. We also found that *MAML2* rearrangement is frequently and largely seen in younger patients, which is similar to that reported by Achcar Rde O et al [19]. Histologically, the rearrangement of *MAML2* is in general recognized to be within cases of low and intermediate grades. Our series included 9 high-grade PMEC cases, all of which were negative for such gene rearrangement. This is in agreement with some previous documents on mucoepidermoid carcinoma of the salivary glands [15], [20], suggesting that a major biological difference between low-/intermediate-grade PMECs and high-grade tumors and other mutational pathways are involved in high-grade tumors. Such feature could be used for grading of PMECs in a molecular manner.

Our study also revealed negative rearrangement for *MAML2* in the total 40 cases of adenosquamous carcinomas. This is in general consistent with the findings reported by Achcar Rde O et al [19]. Our results together with previous data from a smaller series [19] did solidly indicate that *MAML2* rearrangement occurs commonly and is exclusively seen in PMECs, suggesting that it may be a functional marker to facilitate diagnosis and differential diagnosis for this tumor. In particular, certain undecided cases can be identified through detecting the *MAML2* rearrangement on molecular techniques basis, provided that there be evidential prospective studies conducted to validate this methodology.

Moreover, a major new finding of this study is that the presence of *MAML2* rearrangement was associated with longer OS and DFS in PMEC patients. Clearly there exists important biological differences between *MAML2* rearranged tumors and *MAML2* non-rearranged tumors. To date, some clinicopathological prognostic parameters have been defined for PMEC [2]–[4], yet clinical parameters fail to address the underlying biology of the tumor and pathological parameters that are generally assessed subjectively. Thus, further search for the molecular indicators will be necessary, given that the *MAML2* rearrangement is promising in refining clinicopathological prognostic factors for PMEC patients. It should be noted that compared with RT-PCR, FISH studies exclusively exhibited *MAML2* rearrangement, but failed to indicate the fusion partner, which remains necessary to clarify. Nonetheless, considering the high concordance between FISH and RT-PCR [17], [20], and the easy application of FISH method, we and other authors [17] believed that FISH analysis of the *MAML2* gene split might be more useful screening of a favorable subset of mucoepidermoid carcinoma cases.

To further determine the molecular consequences of *MAML2* rearrangement, we studied the potential downstream targets of *MECT1–MAML2* fusion, including Notch targets (HES1) and cAMP/CREB targets (FLT1 and NR4A2) using immunohistochemistry. IHC analysis showed that FLT1 and HES1 were expressed at low levels in *MAML2* rearranged group and contrarily at high levels in *MAML2* non-rearranged group. Similar findings were reported in mucoepidermoid carcinoma of the salivary glands by Behboudi et al using quantitative polymerase chain reaction (qPCR) methods [20]. However, we found no clear difference on the expression of NR4A2 between the two groups. This may be in part due to the relatively low sensitivity of IHC technique compared to qPCR application in previous investigation [20]. Nonetheless, the present study strongly suggests that the *MECT1–MAML2* fusion is a basic event occurred during the process of translocation of 11q21 locus. What's more, our visualization of the potential *MECT1–MAML2* downstream targets in paraffin tumor tissues shows for the first time that FLT1 might be a useful prognostic factor for PMEC cases. Thus, combined molecular detection of *MAML2* rearrangement with FLT1 expression can lead to more reliable predication of the outcomes in patients with such entity.

Finally, *MAML2* rearrangement may be of value for the treatment of PMEC, for some evidences have suggested that *MECT1–MAML2* tend to confer susceptibility to a tyrosine kinase inhibitor (TKI) response. Recent results [21], [22] verified clinical responses to the EGFR TKI (gefitinib) in PMECs in the absence of sensitizing EGFR mutations. Besides, *in vitro* data have shown that the PMEC cell-line (H292), which is EGFR wild-type and carries the t(11;19) and *MECT1–MAML2*, is highly sensitive to gefitinib [22]. Still and clearly, such speculation requires further pre-clinical as well as clinical investigations.

In summary, the current study demonstrated that *MAML2* rearrangement is a frequent event in PMEC and specific to this tumor as compared with primary lung ASC. The presence of *MAML2* rearrangement and absence of FLT1 will confer a favorable clinical outcome for patients with such entity. Combined determination of *MAML2* rearrangement with FLT1 expression status by FISH and IHC, respectively, may represent as important adjunctive diagnostic markers as well as prognostic factors for PMECs. The rearrangement nature of *MAML2* may also allow additional avenues for the clinical management of PMEC. It is worth noting that additional large-scale prospective studies with well-characterized PMECs are needed to further substantiate our findings.
